# Supplementary material for: Complexome profiling of the Chlamydomonas psb28 mutant reveals TEF5 as an early PSII assembly factor
Source: Plant Cell. 2025 Mar 17;37(6):koaf055. doi: 10.1093/plcell/koaf055 (PMC12164590; doi:10.1093/plcell/koaf055)
Supplement: koaf055_Supplementary_Data [file koaf055_supplementary_data.zip › psb28_interactive.zip!/plots/intensity/qualityPlots/peptides/PSBP4_Cre08.g362900.t1.1.html]

### Cre08.g362900.t1.1

Trivial name: PSBP4  
SeqCoverage [median]: 31.30  
Mass: 29.34 kDa  
  
 

| Sequence | MissedCleavage | Mass [Da] | wtIntensity | muIntensity | Score |
| --- | --- | --- | --- | --- | --- |
| LLDQYLTK | 0 | 992.55 | 6161.69 | 3118.94 | 109.86 |
| LQTPTGQYKPDSGALR | 1 | 1730.90 | 1478.66 | 4267.31 | 71.08 |
| NAYAATR | 0 | 765.38 | 1521.79 | 2298.19 | 93.56 |
| SVEDLGSPQDAANR | 0 | 1457.67 | 3276.05 | 3598.51 | 237.53 |
| TADDGKVYYDIAIR | 1 | 1598.79 | 3992.76 | 9035.76 | 116.84 |
| TLSAVLGVANNR | 0 | 1213.68 | 940.82 | 7620.19 | 117.89 |
| YGEIVSANKR | 1 | 1135.60 | 8094.16 | 8853.44 | 115.78 |
| YKSVEDLGSPQDAANR | 1 | 1748.83 | 2301.22 | 1804.64 | 93.51 |
|  |  |  | 27767.2 | 40597.0 |  |
